# Rapid Human Skin Barrier Disruption by Sodium Dodecyl Sulfate and Associated Molecular Mechanisms

**DOI:** 10.1111/all.70390

**Published:** 2026-05-17

**Authors:** Manru Li, Huseyn Babayev, Paolo D'Avino, Can Zeyneloğlu, Ceren Bicer, Duygu Yazici, Yagiz Pat, Per Svedenhag, Nicolas Gaudenzio, Cezmi A. Akdis, Yasutaka Mitamura

**Affiliations:** ^1^ Swiss Institute of Allergy and Asthma Research (SIAF), University of Zurich Davos Switzerland; ^2^ SciBase AB Sundbyberg Sweden; ^3^ University of Toulouse, Inserm, CNRS, Toulouse Institute for Infectious and Inflammatory Diseases (Infinity) Toulouse France; ^4^ Genoskin SAS Toulouse France; ^5^ Christine Kühne – Center for Allergy Research and Education (CK‐CARE) Davos Switzerland; ^6^ Department of Dermatology, Graduate School of Medical Sciences Kyushu University Fukuoka Japan; ^7^ Department of Dermatology National Hospital Organization, Kyushu Medical Center Fukuoka Japan

**Keywords:** electrical impedance spectroscopy, ex vivo human skin, oxidative stress, skin barrier dysfunction, sodium dodecyl sulfate

## Abstract

**Background:**

Epithelial barrier disruption is a hallmark of allergic skin diseases. Sodium dodecyl sulfate (SDS), a surfactant in household cleaning products, is known to impair the barrier.

**Methods:**

We used a physiologically relevant ex vivo human skin combined with real‐time electrical impedance spectroscopy (EIS) to monitor barrier integrity after SDS exposure. Multi‐omics analyses, including RNA sequencing and proximity extension‐based proteomics, characterized molecular responses. Barrier permeability and oxidative stress were evaluated in primary keratinocytes, and the protective effects of N‐acetylcysteine (NAC) and nicotinamide (NAM) were assessed in both air–liquid interface keratinocyte cultures and ex vivo skins.

**Results:**

Even a 1‐min SDS exposure caused a rapid EIS decline, indicating immediate barrier compromise. 5‐min exposures produced dose‐dependent EIS decline with broad suppression of barrier and immune mediators (e.g., CXCL11, MCP4, and HNMT), 6‐h exposure sustained skin barrier loss and associated inflammatory and remodeling programs. Proteomic signatures highlighted AREG, JUN, and ITGA6 can track skin damage, while CST5 and PRDX1 correlated with the preservation. Transcriptomics corroborated these changes, showing up‐regulation of stress and repair programs—endoplasmic reticulum stress, oxidative stress, sphingolipid biosynthesis, and epidermal differentiation pathways. NAC/NAM reduced SDS‐induced reactive oxygen species, cytotoxicity, and permeability in primary keratinocytes, and restored EIS values in ex vivo skin.

**Conclusion:**

Short‐term SDS exposure rapidly disrupts human skin barrier integrity through oxidative stress‐driven suppression of structural/immune mediators and activation of stress/remodeling pathways. NAC and NAM effectively mitigated damages, highlighting antioxidants as preventive interventions for surfactant‐induced skin barrier dysfunction.

## Graphical abstract

Short‐term exposure to SDS rapidly disrupts human skin barrier integrity within minutes, as detected by real‐time electrical impedance spectroscopy. SDS‐induced barrier dysfunction is driven by oxidative stress, leading to suppression of immune/barrier mediators and activation of stress, lipid remodeling, and epidermal differentiation pathways. Antioxidants (NAC and NAM) effectively reduce ROS, restore barrier function, and mitigate SDS‐induced cytotoxicity, highlighting a preventive strategy for surfactant‐induced skin damage. Abbreviations: AP‐1, transcription factor subunit; AREG, amphiregulin; CST5, cystatin D; EIS, electrical impedance spectroscopy; ER, endoplasmic reticulum; ITGA6, integrin alpha‐6; JUN, jun proto‐oncogene; NAC, N‐acetylcysteine; NAM, nicotinamide; PBS, phosphate‐buffered saline; PRDX1, peroxiredoxin 1; ROS, reactive oxygen species; SDS, sodium dodecyl sulfate.

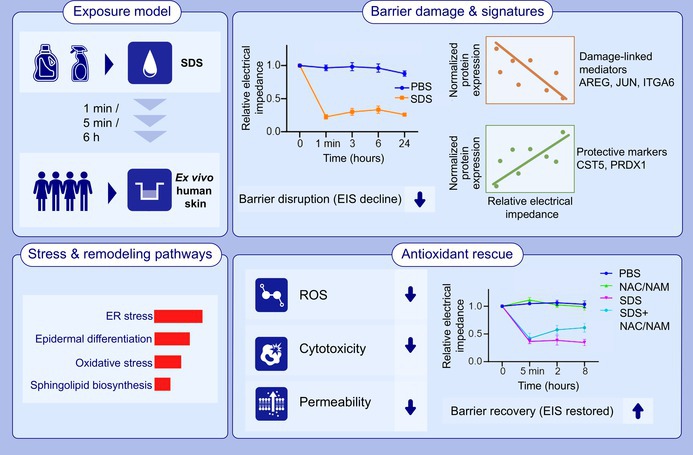

**Abbreviations**
ALI: air–liquid interface
AREG: amphiregulin
CCL2: C–C motif chemokine ligand 2
CCL20: C–C motif chemokine ligand 20
CCL7: C–C motif chemokine ligand 7
CLDN8: claudin‐8
CXCL1: C–X–C motif chemokine ligand 1
CXCL11: C–X–C motif chemokine ligand 11
EIS: electrical impedance spectroscopy
ER: endoplasmic reticulum
FGF19: fibroblast growth factor 19
FGF5: fibroblast growth factor 5
GPX2: glutathione peroxidase 2
IL1A: interleukin‐1 alpha
IL7: interleukin‐7
IRF9: interferon regulatory factor 9
ITGA6: integrin alpha‐6
ITGB6: integrin beta‐6
JUNB: transcription factor JunB
KRT17: keratin 17
LIPN: lipase member N
MMP‐10: matrix metalloproteinase‐10
NAC: N‐acetylcysteine
NAM: nicotinamide
PRDX1: peroxiredoxin 1
ROS: reactive oxygen species
SDS: sodium dodecyl sulfate
SESN2: sestrin‐2
SQSTM1: sequestosome‐1
TRANCE: TNF ligand superfamily member 11 (RANKL)
TSLP: thymic stromal lymphopoietin
uPA: urokinase‐type plasminogen activator

## 1 Introduction

The epithelial barrier of the skin controls the entry of allergens, microbes, and irritants while maintaining homeostasis [1]. Disruption of this barrier not only increases transepidermal water loss (TEWL) but also initiates downstream immune activation and is increasingly recognized as a key factor in the pathogenesis of allergic diseases, including atopic dermatitis and food allergy [2, 3, 4, 5, 6]. It is well known that environmental exposures of modern life, such as air pollution and detergents, cause epithelial damage and inflammation in the exposed tissues.

Among such agents, surfactants contained in household and professional cleaning products are widely implicated in epithelial barrier dysfunction [7, 8, 9, 10, 11, 12, 13, 14, 15, 16]. Even at low concentrations, they activate epithelial cells and perturb stratum corneum and tight junction barrier integrity and lipid disorganization, thereby provoking Th2‐skewed inflammation [7, 17, 18]. Sodium dodecyl sulfate (SDS, also known as sodium lauryl sulfate, SLS) is one of the most ubiquitous anionic detergents used in laundry, dishwashing, and personal care products [19]. Contact with SDS can induce epidermal barrier dysfunction, disrupt junctional proteins, and trigger cutaneous inflammation [3]. Importantly, residual SDS may persist on washed clothes and in house dust, resulting in chronic low‐level skin exposure in daily life [14, 17]. Despite its widespread use, the molecular events triggered in human skin by acute or repeated SDS exposure remain incompletely defined.

Most previous studies have relied on keratinocyte monolayers or reconstructed epidermis, which lack the structural complexity and immune context of human skin. Furthermore, traditional readouts such as TEWL, histology, or endpoint proteomics provide static snapshots and therefore fail to capture the earliest and most transient perturbations of barrier function. Addressing this knowledge gap requires models that preserve the architecture of human skin while enabling a dynamic monitoring of barrier integrity.

Here, we used an ex vivo human skin (NativeSkin, Genoskin SAS, France), which retains the full epidermal–dermal structure and resident immune cells, together with electrical impedance spectroscopy (EIS), a validated noninvasive method for real‐time assessment of barrier function in both in vivo and ex vivo settings [7, 20, 21]. This integrative approach enables physiologically relevant and temporally resolved profiling of skin responses to environmental surfactants. Building on evidence that oxidative stress is a key driver of irritant‐induced injury, we further evaluated two clinically available redox modulators, N‐acetylcysteine (NAC, a cysteine donor and glutathione precursor) and nicotinamide (NAM, the amide form of vitamin B3, a NAD^+^ precursor), for their potential to mitigate SDS‐related barrier disruption.

In this study, we demonstrate that even very short SDS exposures cause immediate impairment of the human skin barrier. Multi‐omics profiling revealed that SDS suppresses barrier‐ and immune‐related mediators while activating stress and tissue remodeling programs. Importantly, we show that oxidative stress is a central mechanism of SDS‐induced injury and that treatment with clinically available antioxidants, NAC and NAM, significantly attenuates cytotoxicity, oxidative damage, and barrier compromise in keratinocyte cultures and ex vivo skins.

## 2 Materials and Methods

### 2.1 Ex Vivo Human Skin Treatment

Ex vivo human skin samples (NativeSkin, GenoSkin SAS) were obtained from adult abdominoplasty donors with written informed consent (*N* = 14, all female, Table S1). Donors had no record of allergies or dermatological disorders and did not use corticosteroids. Full ethical approval for the study protocol was obtained from the French ethical research committee (Comité de Protection des Personnes), and authorization was granted by the French Ministry of Research. Explants were maintained in 2 mL of chemically defined, Xeno‐free medium (GenoSkin) at 37°C and 5% CO_2_.

Skin biopsies were equilibrated at 37°C and treated topically with 60 μL phosphate‐buffered saline (PBS, control) or SDS (Sigma‐Aldrich) at 0.04, 0.2, 1, or 5 mg/mL for 1 min, 5 min (short‐term), or 6 h (long‐term). For recovery experiments, 2 mM NAC (Sigma‐Aldrich) or 6 mM NAM (Sigma‐Aldrich) was applied topically immediately after SDS exposure. Samples were collected at designated time points for downstream analyses.

### 2.2 Electrical Impedance Spectroscopy Measurements

EIS measurement was performed using the Nevisense system (SciBase, Sweden), as previously described [7, 22]. In this study, impedance was measured across 35 frequencies (1 kHz–2.5 MHz) at two depths and three permutations.

The timing of EIS measurements varied by experimental model. The specific time points are shown in the corresponding figures. All measurements were conducted in triplicate and normalized to baseline values.

### 2.3 High‐Throughput Proteomics

Protein expression in ex vivo skin tissue was measured using a proximity extension assay (PEA)–based multiplex platform (Olink, Uppsala, Sweden) with the Target 96 inflammation and immune response panels. Data were reported as normalized protein expression (NPX, log2 scale) after standard preprocessing and quality control with the OlinkAnalyze R package.

Protein differences between treatment groups were tested by *t*‐test with Benjamini–Hochberg correction, and proteins with adjusted *p* < 0.05 were considered significant.

Dose‐dependent protein profiles were clustered using the Mfuzz (v2.68.0) R package [23].

Associations between protein expression and EIS values were examined using Partial Least Squares (PLS) regression (pls v2.8–5), with protein importance assessed by Variable Importance in Projection (VIP) scores.

An all‐vs.‐all Spearman correlation matrix was generated for proteins, SDS dose, and EIS values, and edges with adjusted *p* < 0.05 were retained. A stringent network required an absolute correlation coefficient (|ρ|) ≥ 0.7.

### 2.4 Bulk RNA‐Seq in Ex Vivo Human Skin

Human skin sections were preserved in RNAlater (Agilent), and total RNA was extracted using TRIzol (Invitrogen) and purified with the RNeasy Plus Micro Kit (Qiagen). Samples with RNA integrity numbers (RIN) > 7 were used for stranded mRNA library preparation (Illumina Stranded mRNA Prep). Libraries were sequenced on a NovaSeq 6000 (Illumina) to generate ~30 million paired‐end reads (2 × 100 bp) per sample.

Quality control was performed with FastQC and MultiQC. Reads were aligned to the human genome (GRCh38.p14) using Subread (v2.0.6) [24], and gene‐level quantification was carried out with featureCounts (v2.0.6) [25]. Normalization and differential expression analysis were performed with DESeq2 (v1.48.1) [26], with ComBat‐seq (sva v3.56.0) applied to correct for donor batch effects.

Gene set enrichment was conducted using clusterProfiler (v4.16.0) [27] with Gene Ontology (GO) Biological Process categories. Transcription factor activity was inferred using the viper algorithm (v1.42.0) [28] with regulons from the DoRoThEA database (v1.7.4) [29].

### 2.5 Reactive Oxygen Species Detection

Normal human epidermal keratinocytes (NHEK) (Innoprot, Spain) were cultured as monolayers in EpiLife medium (60 μM Ca^2+^, Gibco) supplemented with Human Keratinocyte Growth Supplement (HKGS). Cells were maintained at 37°C, 5% CO_2_.

Intracellular reactive oxygen species (ROS) levels were measured using the DCFDA/H2DCFDA Cellular ROS Assay Kit (Abcam) according to the manufacturer's protocol. Cells were seeded in black clear‐bottom 96‐well plates, pretreated with 2 mM NAC or 6 mM NAM for 4 h, and then exposed to SDS at the indicated concentrations for an additional 4 h. The optimum doses of NAC and NAM were established in preliminary experiments.

Fluorescence was recorded with a Mithras LB 940 multimode plate reader (excitation/emission 485/535 nm), and representative images were acquired with an EVOS M7000 Imaging System. Experiments were performed in triplicate.

### 2.6 Air‐Liquid Interface Keratinocyte Culture

Air–liquid interface (ALI) culture was established as previously described [30], with minor modifications. Primary normal human epidermal keratinocytes (NHEKs; PromoCell) were cultured in EpiLife medium (60 μM Ca^2+^, Gibco) supplemented with human keratinocyte growth supplement (HKGS). Cells (passages 2–3) were seeded on polyester membrane transwell inserts (0.4 μm pore size, Corning). After 24 h, the medium was supplemented with 1.5 mM CaCl_2_, 10 ng/mL recombinant human FGF‐7 (PeproTech), and 50 μg/mL ascorbic acid (Sigma Aldrich), and ALI conditions were established by removing the apical medium. Cells were maintained under ALI for 14 days to allow differentiation.

On day 14, cells were exposed to SDS in the apical compartment for 5 min. For antioxidant experiments, cells were pretreated for 1 h with 2 mM NAC or 6 mM nicotinamide NAM in the basolateral medium prior to SDS exposure. Cytotoxicity and permeability were subsequently assessed as described.

### 2.7 LDH Cytotoxicity Assay

Cytotoxicity in ALI‐differentiated keratinocytes (day 14) was measured using the CyQUANT LDH Cytotoxicity Assay Kit (Invitrogen) according to the manufacturer's protocol, following SDS exposure to the apical compartment. For antioxidant experiments, cells were pretreated with 2 mM NAC or 6 mM NAM for 1 h in the basolateral medium before SDS treatment. LDH release into the basolateral medium was quantified on a Mithras LB 940 plate reader (absorbance 490/680 nm). Cytotoxicity was calculated relative to maximum lysis controls, and each condition was assayed in triplicate.

### 2.8 FITC‐Dextran Permeability Assay

Barrier integrity was assessed in ALI‐differentiated keratinocytes (day 14) using FITC‐dextran (4 kDa, Sigma‐Aldrich). Following 5 min of SDS exposure with or without antioxidant pretreatment for 1 h, 2 mg/mL of FITC‐dextran was applied apically, and basolateral medium was collected after 24 h. Fluorescence (excitation/emission 485/535 nm) was quantified on a plate reader, and permeability values were normalized to maximum permeability controls according to a previous study [18].

### 2.9 Statistical Analysis

Data were analyzed using GraphPad Prism v9.0. Statistical comparisons between groups were performed using one‐way ANOVA followed by Tukey's multiple comparisons test or the Mann–Whitney U‐test, as appropriate. A *p*‐value < 0.05 was considered statistically significant. Specific statistical methodologies for the high‐throughput proteomics and transcriptomics data are detailed within their respective methods sections (Sections 2.3 and 2.4). Bioinformatics analyses of the RNA‐sequencing and proteomics data are written above in relevant sections.

## 3 Results

### 3.1 SDS Triggers Rapid Skin Barrier Breakdown and Early Proteomic Shifts

**FIGURE 1:**
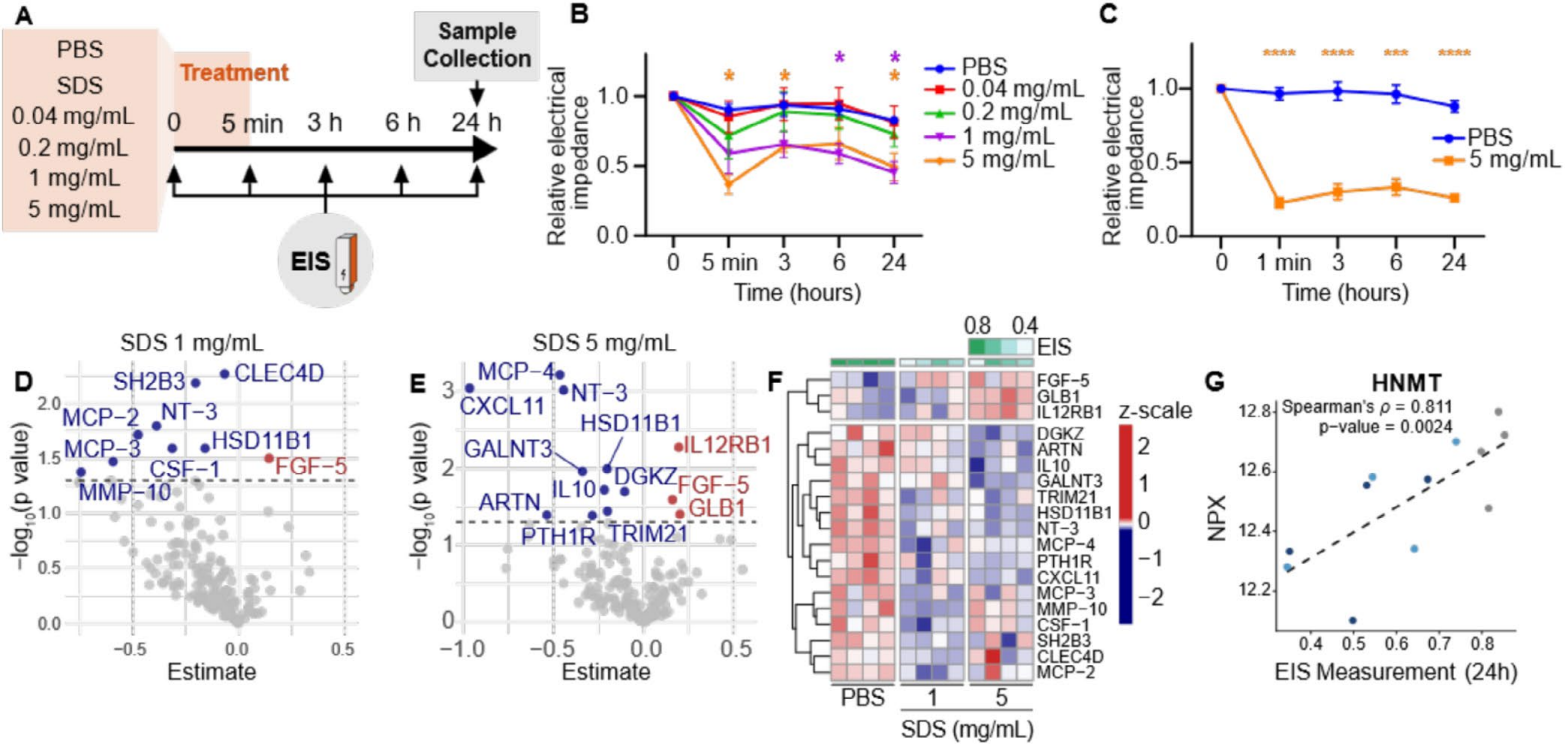
Acute SDS exposure induces rapid skin barrier disruption and suppresses repair‐related protein expression in ex vivo human skin. (A) NativeSkin human skin explants were treated with SDS at different dilutions or PBS (control) for 5 min. EIS was recorded at baseline (0 h) and at 5 min, 3 h, 6 h, and 24 h post‐treatment. (B) EIS values were normalized to baseline for each sample (mean ± SEM, *n* = 4 samples from 3 independent donors). (C) A 1‐min exposure to 5 mg/mL SDS decreased EIS at multiple time points compared to PBS (*n* = 6 samples from 3 independent donors). (D, E) Volcano plots of differentially expressed proteins from skin lysates 24 h after 5‐min SDS exposure at 1 mg/mL (D) or 5 mg/mL (E) by proximity extension combined with proteomics; red and blue indicate upregulated and downregulated proteins (*p* < 0.05). (F) Heatmap of differentially expressed proteins showing dose‐dependent expression changes. The top green annotation indicates Spearman correlations between SDS concentration and donor EIS values. (G) Spearman correlation between HNMT levels (NPX) and EIS at 24 h (*r* = 0.811, *p* = 0.0024). Statistical significance: One‐way ANOVA; *p < 0.05, ***p < 0.001, ****p < 0.0001.

To assess the acute effects of SDS, NativeSkin samples were exposed to graded SDS concentrations, with PBS as a control. Barrier integrity was assessed by EIS at baseline (0 h) and up to 24 h (Figure 1A). Even the highest concentration, 5 mg/mL (0.5% w/v), is less than the concentrations found in many personal‐care products (1%–5%) and detergents (5%–15%). Doses as low as 1 mg/mL showed a significant epithelial barrier‐damaging effect in the range of a detergent remnant on the cloths and bed sheets after a 40°C medium time laundry wash [17].

EIS showed that just 5 min of SDS exposure caused a dose‐dependent barrier dysfunction, evidenced by reduced impedance (Figure 1B). Remarkably, even 1 min of exposure to 5 mg/mL SDS resulted in a robust and statistically significant impedance drop at multiple time points up to 24 h (Figure 1C), indicating that barrier disruption initiates within minutes of exposure.

Targeted proteomic profiling at 24 h showed concentration‐dependent alterations in protein expression. At 1 mg/mL, only fibroblast growth factor‐5 (FGF‐5) was significantly upregulated (Figure 1D). At 5 mg/mL, additional proteins, including interleukin‐12/23 receptor β1 (IL12RB1) and β‐galactosidase (GLB1), were induced, while numerous immune‐ and repair‐related mediators were suppressed (Figure 1E). The heatmap illustrates these shifts in control, 1 mg/mL, and 5 mg/mL samples, alongside donor EIS values (top track) (Figure 1F).

Spearman correlation analysis revealed that histamine N‐methyltransferase (HNMT) expression strongly correlated with EIS values at 24 h (Figure 1G). Additional proteins positively associated with barrier integrity included monocyte chemotactic proteins (MCP‐2/3/4), C‐X‐C motif chemokine ligand 11 (CXCL11), neurotrophin‐3 (NT‐3), SH2B adaptor protein 3 (SH2B3), and tripartite motif‐containing protein 21 (TRIM21) (Figure S1).

### 3.2 Prolonged SDS Exposure Drives Persistent Barrier Loss and Distinct Dose‐Dependent Molecular Response Patterns

**FIGURE 2:**
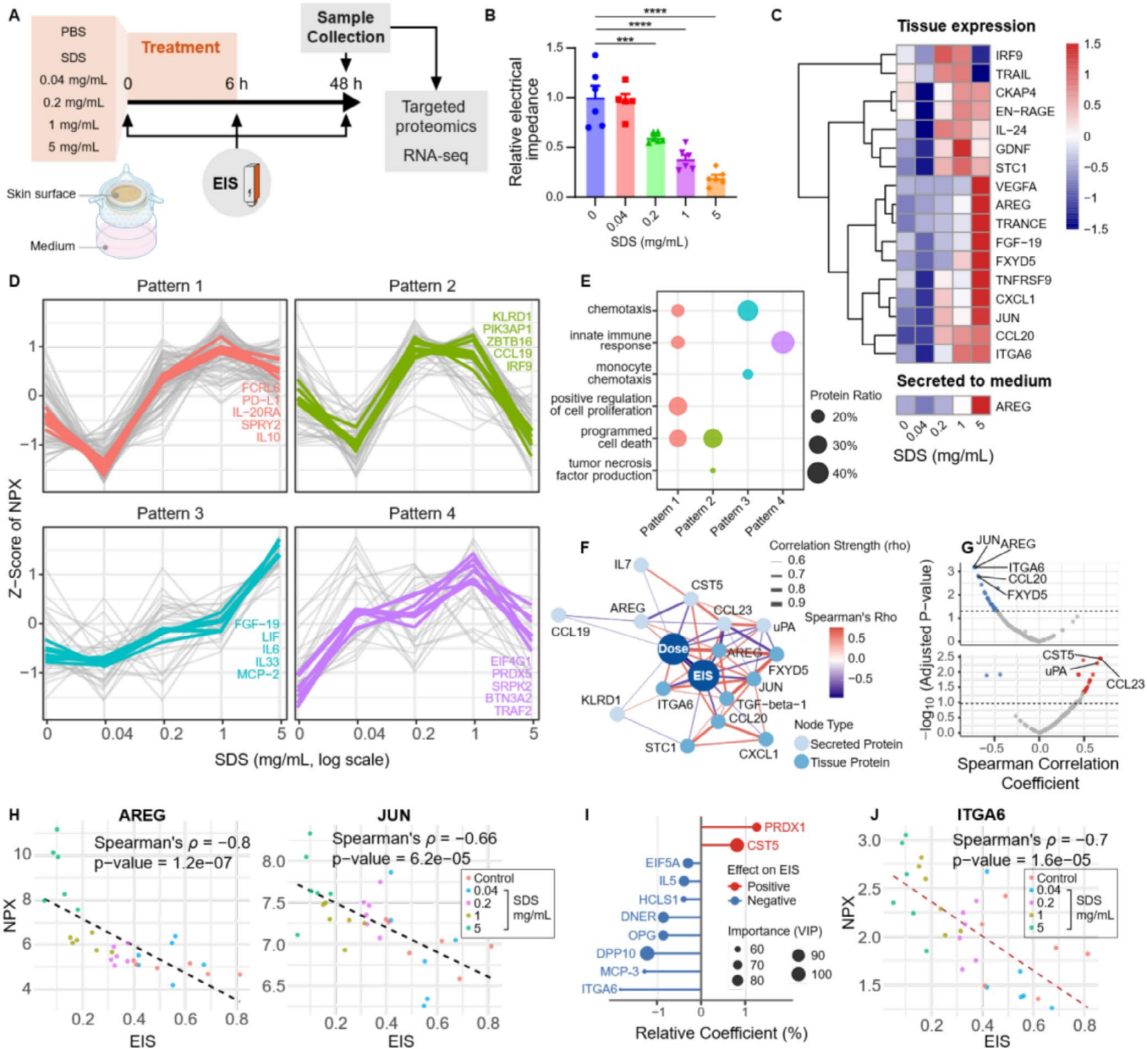
Prolonged SDS exposure induces severe barrier dysfunction and dose‐dependent proteomic signatures that distinguish protective vs. damage‐linked mediators in ex vivo human skin. (A) Ex vivo human skin (NativeSkin) samples were treated with 0.04, 0.2, 1, or 5 mg/mL SDS for 6 h. EIS was measured up to 48 h after the treatment. (B) Relative electrical impedance at 48 h shows dose‐dependent barrier loss (mean ± SEM, *n* = 6 samples from 3 independent donors). (C) Heatmaps of significantly altered proteins 48 h after SDS exposure, measured by Olink Immune Response and Inflammation panels, showing dose‐dependent changes in tissue expression (top) and secretion into medium (bottom). (D) Clustered dose response trajectories identified four expression patterns. Representative proteins are shown to illustrate characteristic trajectories. (E) Pathway enrichment analysis of each trajectory pattern. (F) Correlation network linking protein expression, EIS, and SDS dose; edge color and thickness indicate correlation direction and strength (Spearman's ρ); node color distinguishes secreted (light blue) and tissue‐localized (dark blue) proteins. (G) Significance effect plots of Spearman correlations between analyte levels and EIS; top panel: Negative correlations (blue), bottom panel: Positive correlations (red). (Spearman's ρ > 0.5 and adjusted *p* < 0.05). (H) Scatter plots show correlations between EIS values measured 48 h after SDS exposure and protein expression levels (normalized protein expression; NPX, log2 scale) in skin tissue lysates at the same time point. Spearman correlation coefficient (ρ) and *p*‐value are indicated. Dose–response curves of AREG and JUN showing inverse correlation with EIS values. (I) Partial least squares (PLS) regression with variable importance in projection (VIP) scores. (J) Dose–response curve of ITGA6 expression confirming its inverse relationship with EIS values.

Residual surfactants have been detected in rinse water and household dust following regular use of cleaning products. To mimic prolonged environmental surfactant exposure, ex vivo human skin explants were topically treated with SDS (0.04, 0.2, 1, and 5 mg/mL) for 6 h, and after a medium change, followed by 42 h for a recovery period (Figure 2A). Barrier function was assessed using EIS, and samples were collected at 48 h post‐treatment for transcriptomic and targeted proteomic analysis via proximity extension assay and RNA‐seq. EIS at 48 h post‐SDS exposure revealed a sustained, dose‐dependent reduction in skin barrier integrity, with significant impairment from 0.2 mg/mL and a maximal ~70% loss at 5 mg/mL (Figure 2B), indicating a severe barrier impairment.

Targeted proteomic profiling at 48 h revealed a predominantly dose‐dependent upregulation of immune and repair mediators, including VEGFA, AREG, FGF19, FXYD5, CXCL1, JUN, CCL20, and ITGA6 (Figure 2C). In contrast, proteins such as IRF9 and TRAIL/TNFSF10 displayed biphasic expression, increasing at low to intermediate doses but suppressed at 5 mg/mL. This biphasic pattern observed for IRF9 and TRAIL was further contextualized by classifying all concentration‐dependent expression trajectories into four major patterns (Figure 2D). Pattern 1 encompassed proteins that were upregulated at 0.2–1 mg/mL with only a modest decline at 5 mg/mL; representative members include FCRL6, PD‐L1, IL‐20RA, SPRY2, and IL‐10. Pattern 2 comprised proteins that also rose at intermediate doses, but were strongly suppressed at 5 mg/mL. Examples include KLRD1, PIK3AP1, ZBTB16, CCL19, and IRF9. Pattern 3 displayed a monotonic and linear increase across all tested concentrations (0.04–5 mg/mL), making it the only cluster with sustained dose‐dependent upregulation; e.g., FGF19, LIF, IL‐6, IL‐33, and MCP‐2/CCL8. Pattern 4 included proteins that were induced at the lowest concentration (0.04 mg/mL), further elevated at 0.2–1 mg/mL, and then sharply downregulated at 5 mg/mL; representative examples include EIF4G1, PRDX5, SRPK2, BTN3A2, and TRAF2. In Figure 2D, we annotate a small set of representative proteins to illustrate the archetypal trajectory shapes, whereas the pathway enrichment in Figure 2E is derived from the complete protein membership of each pattern. The full membership of each pattern and their dose‐dependent profiles are provided in Figure S2.

To better understand the biological programs underlying these patterns, we performed pathway enrichment analysis (Figure 2E). Pattern 1 was linked to programmed cell death, proliferation control, innate immune response, and chemotaxis, suggesting some degree of immune regulation under stress conditions. Pattern 2 was enriched for programmed cell death with additional association to TNF production, reflecting transient engagement of stress‐linked immune pathways that diminish under maximal SDS exposure, probably due to cell death. Pattern 3 was dominated by chemotaxis proteins consistent with a progressive cytokine‐driven immune cell influx across concentrations. Pattern 4 was primarily enriched for the innate immune response, indicating a rapid but fragile activation of fundamental defense modules that collapses at the highest SDS concentration.

### 3.3 Barrier‐Associated Proteomic Profiles Distinguish Protective and Damage‐Linked Mediators in SDS‐Exposed Skin

A correlation network integrating SDS dose, EIS values, and protein expression was constructed using Spearman's rho (Figure 2F). In this network, proteins such as AREG, CCL20, CXCL1, and JUN correlated positively with SDS concentration and negatively with EIS, whereas cystatin D (CST5), C–C motif chemokine ligand 23 (CCL23), and urokinase‐type plasminogen activator (uPA) correlated positively with EIS and negatively with SDS.

Univariate analysis (Figure 2G) further identified two contrasting sets of markers. Negatively associated proteins included JUN, AREG, ITGA6, CCL20, and FXYD5, showing higher expression with lower EIS. Positively associated proteins included CST5, uPA, and CCL23, showing higher expression with higher EIS. Figure 2H shows progressive induction of AREG and JUN with increasing SDS concentrations, consistent with their negative correlation with EIS. Beyond traditional univariate correlations, partial least squares (PLS) regression analysis (Figure 2I) was utilized to capture the complex interplay between protein profiles and EIS values. By integrating the expression patterns of all measured proteins, this model identified proteins most predictive of barrier status. PRDX1 and CST5 were strongly positively associated with EIS, whereas ITGA6, MCP‐3, OPG, DNER, and IL‐5 were negatively associated. This was corroborated by the observation that ITGA6 levels rose progressively as SDS exposure impaired the skin barrier (Figure 2J), confirming its role as a sensitive indicator of tissue damage. Supplementary analyses stratified these associations by compartment, separating tissue‐derived proteins (Figure S3) from secreted proteins in culture medium (Figure S4).

### 3.4 SDS Induces Dose‐Dependent Transcriptomic Remodeling Across Stress, Lipid, and Differentiation Pathways

**FIGURE 3:**
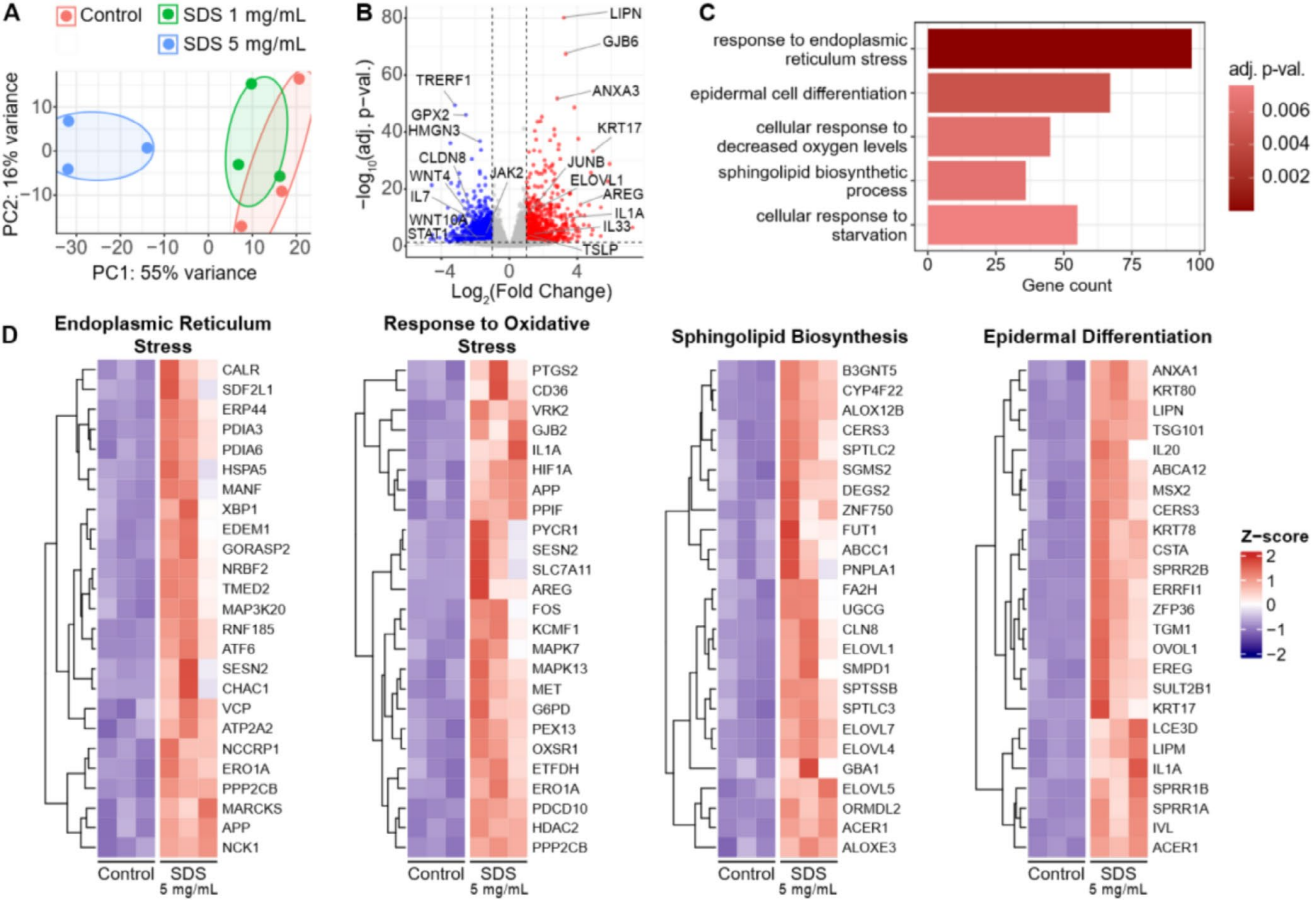
SDS triggers dose‐dependent stress responses, lipid biosynthesis, and barrier‐associated transcriptional changes in ex vivo human skin. (A) Principal Component Analysis (PCA) of RNA‐seq data from PBS, 1 mg/mL SDS, and 5 mg/mL SDS groups (6 h exposure, collection at 48 h after the exposure). (B) Volcano plot of differentially expressed genes (DEGs) in 5 mg/mL SDS vs. PBS; selected upregulated genes red and downregulated genes (blue) are labeled (adjusted *p* < 0.05). (C) Enrichment of upregulated pathways following SDS exposure. GO BP analysis identifies significantly upregulated pathways with the number of differentially expressed genes in the 0.05 mg/mL SDS‐treated group compared to the PBS control. (D) Heatmaps (Z‐score) of representative DEGs in four modules: ER stress, oxidative stress, sphingolipid biosynthesis, and epidermal differentiation (adjusted *p* < 0.05).

RNA‐seq was performed on ex vivo human skin treated with PBS, 1 mg/mL SDS, or 5 mg/mL SDS for 6 h, with recovery to 48 h. Principal component analysis (PCA) showed clear separation of SDS‐treated samples from controls, with the 5 mg/mL group showing the largest divergence along principal component 1 (PC1, 55% variance) and the 1 mg/mL group positioned intermediately along PC2 (16% variance), indicating a dose‐dependent global transcriptomic shift (Figure 3A). Differential expression analysis identified 1697 upregulated and 1673 downregulated transcripts at 5 mg/mL compared to control, PBS (adjusted *p* < 0.05) (Figure 3B). Prominent upregulated genes included *LIPN*, *GJB6*, *AREG*, *KRT17*, *JUNB*, *ELOVL1*, *TSLP*, *IL1A*, and *IL33*. Whereas downregulated genes included *TRERF1, GPX2, HMGN3, CLDN8, WNT4, WNT10A*, and *IL7*. Enrichment analysis of GO Biological Processes highlighted major pathways impacted by SDS exposure (Figure 3C). Upregulated genes were enriched in response to endoplasmic reticulum (ER) stress, epidermal cell differentiation, cellular response to decreased oxygen levels, and sphingolipid biosynthetic process. In contrast, downregulated genes were enriched in processes related to defense response to virus, response to virus, DNA‐templated DNA replication, and response to toxic substance (Figure S5). Pathway‐focused heatmaps revealed coherent transcriptional responses in four functional modules, which are listed as the ER stress/unfolded protein response (UPR) (e.g., *HSPA5*), oxidative stress (e.g., *SESN2*), sphingolipid biosynthesis (e.g., *UGCG, CERS3, ELOVL1*), and epidermal differentiation (*KRT17, SPRR1A, SPRR1B, TGM1*) (Figure 3D).

### 3.5 NAM And NAC Attenuate SDS‐Induced Oxidative Stress and Barrier Disruption

**FIGURE 4:**
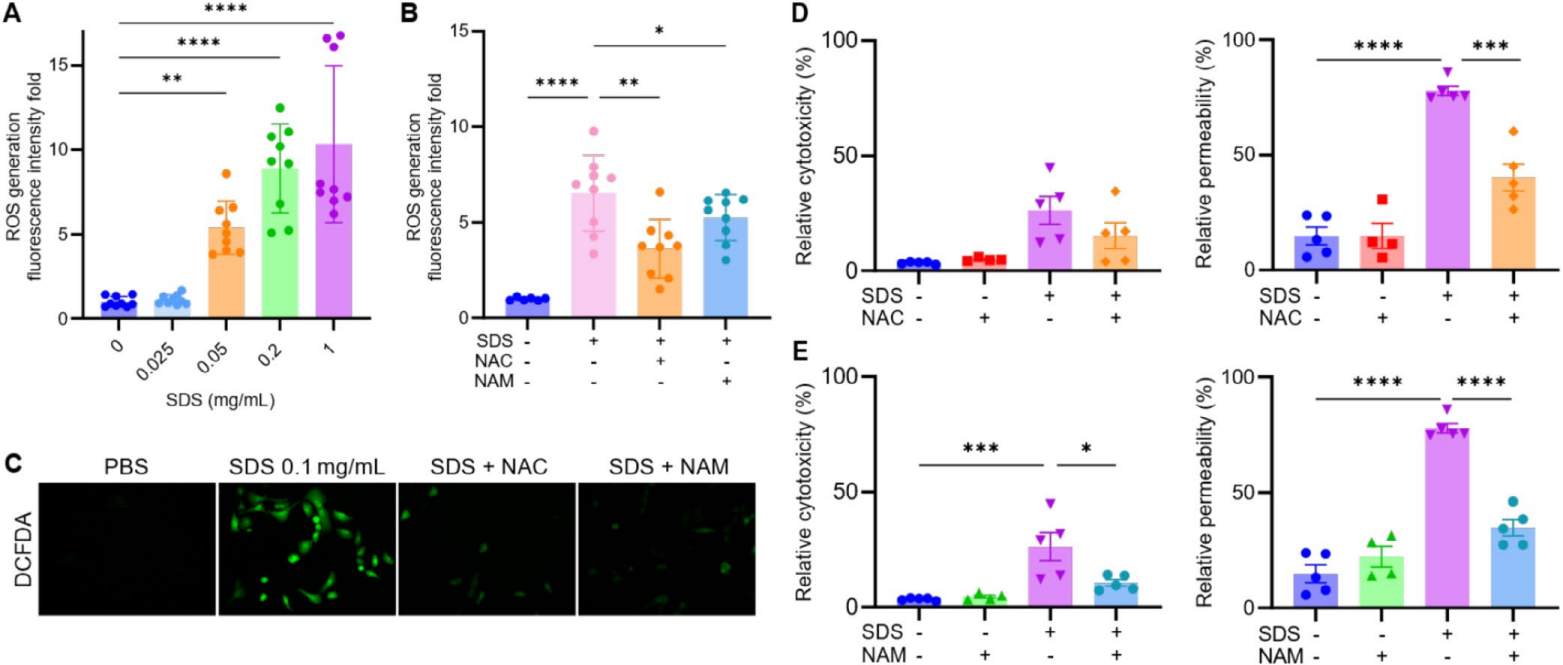
Antioxidant treatment attenuates SDS‐induced ROS production and barrier disruption in both monolayer and ALI keratinocyte models. (A) Relative intracellular ROS levels in monolayer NHEKs at 4 h after SDS exposure (*n* = 9). (B) Pretreatment with 2 mM N‐acetylcysteine (NAC) or 6 mM nicotinamide (NAM) for 4 h before 0.1 mg/mL SDS reduced reactive oxidative species (ROS) accumulation (*n* = 3). (C) Representative live‐cell fluorescence images showing ROS in NHEKs with or without antioxidant pretreatment prior to SDS exposure. (D, E) Relative cytotoxicity (LDH release; left) and barrier permeability (FITC‐dextran flux; right) in ALI‐differentiated NHEKs, pretreatment with NAC (2 mM, 1 h) (D) or NAM (6 mM, 1 h) (E) before SDS exposure (1 mg/mL, 5 min) (*n* = 5). Statistical significance: One‐way ANOVA; *p < 0.05, **p < 0.01, ***p < 0.001, ****p < 0.0001.

Since SDS exposure induced oxidative stress and impaired barrier function, we next tested whether antioxidant treatment could mitigate these effects. Cellular ROS assays in keratinocyte monolayers showed a dose‐dependent increase in ROS, with significant elevations at concentrations of 0.05 mg/mL and more than above (Figure 4A). These findings confirm that SDS induces oxidative stress in keratinocytes in a concentration‐dependent manner. Pretreatment with NAC (2 mM) or NAM (6 mM) for 4 h significantly reduced ROS accumulation after 0.1 mg/mL SDS exposure (Figure 4B). Parallel control experiments confirmed that these concentrations of NAC and NAM were not cytotoxic (Figure S6). Fluorescence microscopy further demonstrated intracellular ROS induction by SDS, which was markedly reduced in NAC‐ or NAM‐pretreated keratinocytes (Figure 4C). To examine consequences for barrier integrity, we used the ALI keratinocyte model. SDS (1 mg/mL) treatment significantly increased cytotoxicity and FITC‐dextran permeability, indicating barrier disruption. NAC and NAM effectively reduced SDS‐induced cytotoxicity and barrier disruption. NAC and NAM significantly reduced SDS‐induced cytotoxicity and alleviated the increase in barrier permeability (Figure 4D,E), confirming the epithelial protective effect of both substances.

### 3.6 Antioxidant Interventions Promote Barrier Recovery Following SDS‐Induced Damage in Human Skin

**FIGURE 5:**
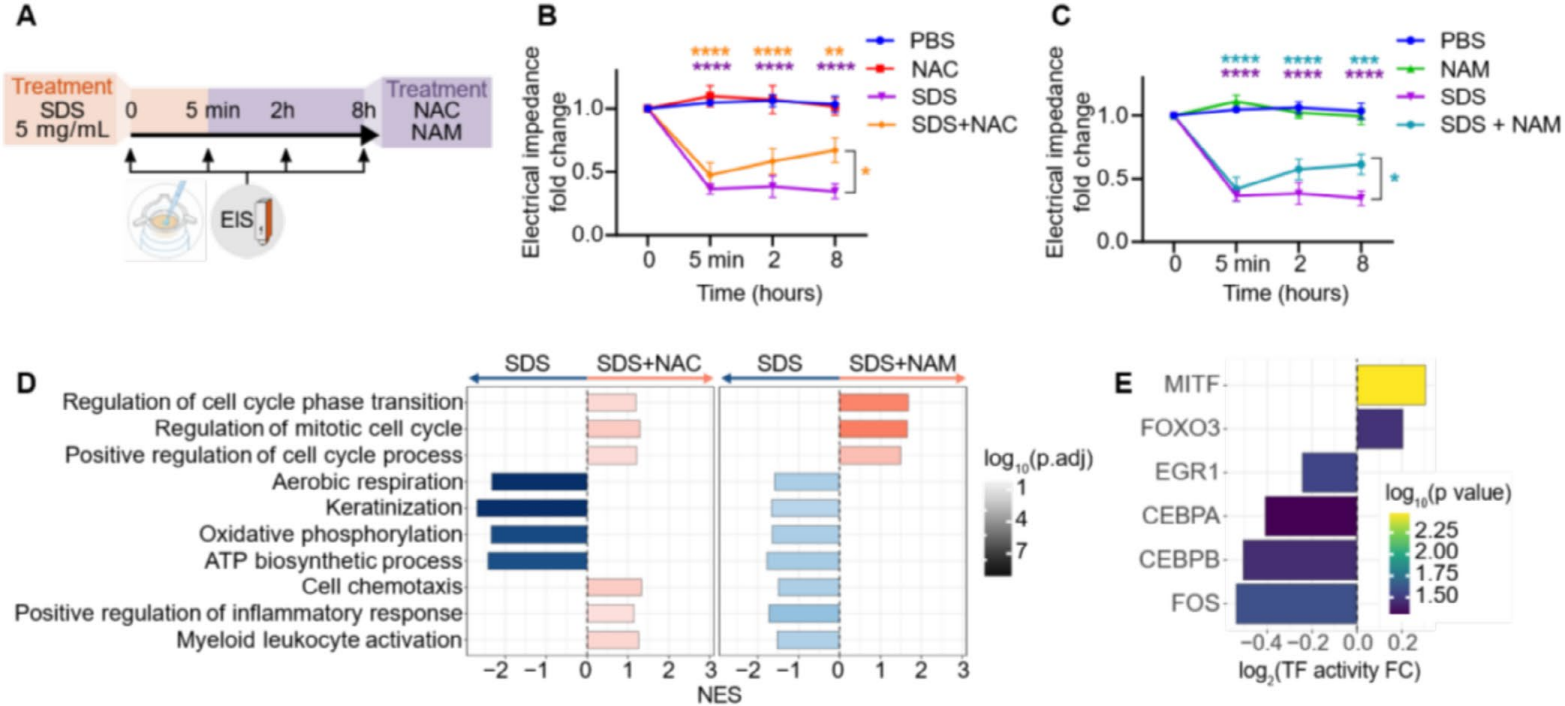
NAC and NAM promote recovery of skin barrier function following SDS‐induced damage in ex vivo human skin. (A) Ex vivo human skin (NativeSkin) samples were exposed to SDS (5 mg/mL) for 5 min, followed by topical application of 2 mM N‐acetylcysteine (NAC) or 6 mM nicotinamide (NAM) for 8 h. EIS measurements were recorded at 0, 5 min, 2 h, and 8 h post‐exposure. (B, C) EIS measurements were performed at baseline (0 h) and after SDS exposure (5 mg/mL, 5 min) with or without subsequent antioxidant application (2 mM NAC or 6 mM NAM) at the indicated time points. Data were normalized to the 0 h value of each sample. One‐way ANOVA was used for comparisons with SDS‐only at each time point; *p < 0.05, ***p < 0.001, ****p < 0.0001. (D) Gene‐set enrichment (GO Biological Process) comparing SDS to SDS + NAC (left) and SDS to SDS + NAM (right) highlights antioxidant‐associated shifts within leukocyte activation/chemotaxis, oxidative phosphorylation/ATP biosynthesis, keratinization, and cell‐cycle control. Bars show normalized enrichment score (NES); dot size encodes –log_10_(adjusted *p*). The brightness scale was used for consistency across both panels. (E) Transcriptional factor (TF)‐activity inference (VIPER using DoRothEA regulons) identifies significant modulation of immediate‐early and stress/metabolic regulators in SDS + NAM compared to SDS alone; bars indicate log_2_ fold‐change in activity and dots show –log_10_(*p*).

Human ex vivo skins were exposed to 5 mg/mL SDS for 5 min, followed by treatment with NAC or NAM (Figure 5A). SDS caused an immediate impedance drop, indicating barrier disruption. Treatment with 2 mM NAC led to a partial recovery, with significantly higher impedance than SDS alone at 8 h (Figure 5B). 6 mM NAM also enabled the recovery of skin impedance (Figure 5C), with significant increases compared to SDS alone at 8 h. The sole usage of PBS, NAC, and NAM did not change the skin barrier; rather, it maintained a stable impedance throughout.

RNA‐seq was performed on these ex vivo human skin samples as well. Pathway analyses were conducted as explicit comparisons of SDS + antioxidants compared to SDS alone (Figure 5D). Proliferation and cell‐cycle programs were significantly enriched in both comparisons, with larger normalized enrichment scores (NES) in the SDS + NAM compared to SDS alone, indicating a more pronounced pro‐proliferative response under NAM. On the other side, oxidative phosphorylation and aerobic‐respiration pathways were comparatively reduced (negative NES) relative to SDS alone in both contrasts. Inflammation‐related signals were comparable in magnitude to the proliferative and metabolic shifts and differed by agent, with SDS + NAC compared to SDS showing increased enrichment of inflammation‐ and chemotaxis‐associated terms (positive regulation of inflammatory response, myeloid leukocyte activation, cell chemotaxis), whereas SDS + NAM compared to SDS showed decreased enrichment of the same pathways. Transcription‐factor activity inference for the SDS + NAM compared to SDS showed decreased activity of immediate‐early/stress regulators (FOS, CEBPA/CEBPB, EGR1) alongside increased activity of FOXO3 and MITF, a pattern consistent with reduced inflammatory drive and enhanced metabolic regulation and epidermal differentiation (Figure 5E). No transcription‐factor activity changes reached significance (*p* < 0.05) for SDS + NAC compared to SDS.

## 4 Discussion

The present study demonstrates a dynamic view of the molecular mechanisms underlying detergent‐induced skin barrier disruption and recovery in human ex vivo skin. Using a combination of biophysical measurements and multi‐omics analyses, we reveal that even acute exposure to SDS rapidly compromises skin barrier function and transiently suppresses epithelial immune‐defense mediators. We identified key molecular pathways and biomarkers associated with barrier damage and restoration, including oxidative stress, lipid synthesis, and keratinocyte differentiation. Antioxidant interventions with NAC and NAM efficiently mitigated SDS‐induced skin injury and preserved human skin barrier function.

Acute exposure to SDS rapidly compromised the physical barrier integrity, as reflected by the decline in EIS values within minutes. The very rapid effects within minutes would be due to rapid surface tension changes and stretching of the tissues. However, the intermediate‐term findings elucidate the molecular mechanisms of SDS‐induced barrier dysfunction and highlight potential pathways for the prevention and treatment of irritant contact dermatitis induced by detergents. The proteomic profiling indicated a broad downregulation of immune and repair mediators. The suppression of chemokines such as CXCL11 and MCP‐2/3/4, the neurotrophic factor NT‐3, and remodeling enzyme matrix metalloproteinase‐10 (MMP‐10) indicates a fundamental role in coordinating immune recruitment and tissue turnover [31, 32, 33]. The widespread reduction of these molecules suggests that SDS‐induced damage not only destabilizes the stratum corneum structurally but also impairs the ability to recruit innate immune cells and initiate repair responses. HNMT is a main enzyme responsible for histamine inactivation, and histamine is known to impair keratinocyte differentiation and barrier function [34]. Among the identified proteins, HNMT showed a positive correlation with EIS values, implicating histamine metabolism in the maintenance of barrier stability. Thus, efficient histamine clearance via HNMT may preserve functional skin barrier integrity. Moreover, other proteins positively associated with intact barrier states, including CXCL11, NT‐3, Flt3L, uPA, and TRIM21, support the concept that the maintenance of skin barrier relies on the regulation of immune recruitment and tissue homeostasis. The reduction of these mediators under high‐dose SDS suggests that excessive surfactant exposure not only causes structural damage but also suppresses the epidermis' innate defense and recovery capacity. These findings underscore the vulnerability of human skin to short‐term (5 min) SDS contact and highlight candidate molecular markers, including HNMT and CXCL11, that link proteomic changes to functional outcomes leading to barrier damage.

As exposure time increased and recovery progressed, we observed a shift from suppression to induction of inflammatory and regenerative mediators. In contrast to a sustained shutdown following acute exposure, proteins such as AREG, CXCL1, and TRANCE (RANKL) were markedly elevated, suggesting activation of epidermal wound repair programs. AREG, an EGF‐receptor ligand, plays a pivotal role in promoting tissue repair and regeneration [35]. CXCL1 is a potent neutrophil chemoattractant indicative of acute inflammation, while TRANCE (RANKL) can engage RANK on various cells to expand regulatory T cells and dampen excessive inflammation [36]. Together, these results suggest that stronger and longer stimulation initiates keratinocyte repair programs and modulates skin homeostasis.

Our trajectory and cluster analyses further identified that SDS elicits four distinct patterns of protein expression across the concentration gradient. One cluster increased monotonically with SDS dose, enriched in chemokine and proinflammatory mediators such as IL6 and IL33, representing transient activation that cannot be maintained even under severe stress. In contrast, another cluster displayed a rise at intermediate doses followed by collapse at the highest dose, representing transient activation followed by suppression by feedback inhibition or cell death. The enriched pathways derived from the proteins of each pattern showed that chemotaxis is activated along with the concentration of SDS. Programmed cell death and innate immune response are suppressed in the maximal dose of SDS at this time point. Network correlation further emphasized opposing molecular signatures linked to barrier status. Proteins such as AREG, CCL20, CXCL1, and JUN were positively correlated with SDS concentrations and negatively correlated with EIS, identifying them as markers of ongoing skin barrier impairment and active skin inflammation. AREG is strongly released after epithelial injury and plays a major role in tissue regeneration [37]. JUN acts as a key transcription factor driving keratinocyte activation during injury [38, 39, 40]. Furthermore, PLS regression analysis identified that antioxidant and homeostatic mediators, including cystatin D2 (CST5) and peroxiredoxin1 (PRDX1), were enriched in samples with preserved barrier integrity. CST5 is a cysteine protease inhibitor, and its loss of function in skin impairs cornification and compromises barrier integrity [41, 42]. Similarly, PRDX1 mitigates oxidative stress through scavenging ROS [43]. The reciprocal relationship between these protective factors and barrier integrity suggests that oxidative and proteolytic balance is essential in sustaining epidermal resilience. Meanwhile, the inverse correlation of ITGA6 and other stress markers with EIS underscores their association with injury. ITGA6, a key component of the basal keratinocyte hemidesmosomal integrin complex, is known to be upregulated during re‐epithelialization and basement membrane remodeling in wound healing [44, 45]. These findings highlight AREG, JUN, and ITGA6 as potential biomarkers of epithelial damage, and CST5 and PRDX1 as indicators of protection, together providing candidate signatures that may predict barrier vulnerability or resilience following irritant exposure.

The transcriptomic profiling provided deeper insights into the cellular response during SDS‐induced stress. The most striking finding was the dual activation of unfolded protein response (UPR) and oxidative stress pathways upon SDS exposure. The strong induction of *HSPA5*, encoding the ER luminal chaperone BiP, and stress‐inducible transcription factors such as *XBP1* and *ATF6* confirms engagement of UPR, indicative of ER protein misfolding [46, 47]. SDS also elicited a marked oxidative/metabolic stress response, as evidenced by *SESN2* induction. ROS and metabolic stress through NRF2 and ATF4 induce SESN2 [48] and act to activate AMPK and stimulate antioxidant defenses. The strong *SESN2* signal thus indicates that SDS induces a redox imbalance in keratinocytes, linking the chemical insult to downstream oxidative stress pathways. In parallel, SDS markedly upregulated genes for epidermal lipid barrier synthesis, including *UGCG*, *CERS3*, and *ELOVL1*. Their upregulation likely represents an adaptive attempt to rebuild the stratum corneum after detergent‐induced lipid extraction [49]. Consistent with these changes, terminal differentiation markers were also elevated. Keratins, such as *KRT17* and the small proline‐rich protein (SPRR) family members (*SPRR1A/B*), are upregulated in hyperproliferative or stress‐activated keratinocytes, where they help reinforce the cornified envelope and provide structural resilience [50, 51]. These results suggest that human skin responds to SDS‐induced stress by simultaneously activating ER stress, oxidative stress, lipid production, and epidermal differentiation.

Finally, the importance of oxidative stress in SDS‐induced skin barrier damage was verified by antioxidant intervention experiments. Pretreatment with the antioxidants NAC or NAM significantly alleviated the SDS‐induced surge in ROS and preserved barrier function. NAC is a classic ROS scavenger and glutathione precursor known to protect cells from a variety of oxidative insults [52]. NAM has multifaceted anti‐inflammatory and antioxidant properties: It enhances nicotinamide adenine dinucleotide (NAD^+^) pools, promoting DNA repair and energy‐dependent healing processes, and barrier lipid synthesis [53, 54]. Consistently, NAC and NAM quenched the excess free radicals generated in cells after treatment with SDS and ameliorated cytotoxicity and barrier impairment induced by SDS. In an ex vivo skin model, post‐exposure topical application of NAC or NAM restored the downregulated EIS induced by SDS over 8 h. At the pathway level, both treatments after SDS exposure showed enrichment of proliferation and cell‐cycle programs, accompanied by a transient reduction in oxidative phosphorylation/aerobic respiration. Consistent with this, NAM reduced activity of immediate‐early/stress regulators such as FOS, CEBPA/CEBPB, and EGR1, while increasing FOXO3 and MITF. No significant TF‐activity changes were detected by NAC treatment.

Clinical and preclinical studies have demonstrated that niacinamide reduces UV‐ and chemical‐induced DNA damage and oxidative stress in skin, while improving barrier metrics such as stratum corneum hydration and trans‐epidermal water loss [55, 56]. In our model, NAM pretreatment likely improved keratinocyte metabolic resilience and antioxidant capacity, thus attenuating SDS's damaging effects. By neutralizing ROS and supporting the skin's repair pathways, agents like NAC and niacinamide could help maintain barrier integrity in the face of chemical stresses commonly encountered in consumer products or occupational exposures [57].

This study has several limitations that should be considered. The use of an ex vivo human skin, while preserving tissue architecture, lacks systemic circulation and immune cell trafficking, which may not fully replicate the in vivo inflammatory response. Furthermore, our multi‐omics analyses are correlational in nature, identifying strong associations between molecular changes and barrier function but not establishing causality. Mechanistic validation of oxidative stress as a key driver was performed in simplified keratinocyte monocultures and air–liquid interface models, and these findings are extrapolated to the more complex full‐thickness skin explant system. The number of skin donors used for native skin experiments was limited, which may affect the generalizability of the results across a wider population. Therefore, while this work identifies potential biomarkers and mechanisms of skin barrier injury vs. protection and maintenance of integrity, further functional validation and in vivo studies are required to confirm these findings.

In conclusion, our findings delineate a mechanistic framework in which oxidative stress serves as a central mediator linking chemical exposure to barrier dysfunction and inflammatory signaling. SDS induces excessive ROS production and damages redox balance, leading to ER stress, protein misfolding, and subsequent suppression of immune homeostasis. The identification of key markers such as HNMT, AREG, ITGA6, CST5, and PRDX5 provides a molecular signature distinguishing phases of injury and repair, offering potential biomarkers for assessing irritant susceptibility or therapeutic efficacy. The antioxidants, NAC and NAM, can effectively protect and restore barrier function. This work advances the understanding of epithelial stress responses to chemical irritants and provides a mechanistic foundation for developing protective interventions against irritant contact dermatitis under chemical stress.

## Author Contributions

Conceptualization: Cezmi A. Akdis and Yasutaka Mitamura. Methodology: Manru Li, Paolo D'Avino, Yagız Pat, Huseyn Babayev, and Yasutaka Mitamura. Investigation: Manru Li and Yasutaka Mitamura. Visualization: Manru Li, Huseyn Babayev, and Yasutaka Mitamura. Funding acquisition: Cezmi A. Akdis. Project administration: Manru Li and Yasutaka Mitamura. Supervision: Nicolas Gaudenzio, Can Zeyneloğlu, Ceren Bicer, Duygu Yazici, Per Svedenhag, Cezmi A. Akdis, and Yasutaka Mitamura. Writing – original draft: Manru Li and Yasutaka Mitamura. Writing – review and editing: Cezmi A. Akdis and Yasutaka Mitamura.

## Funding

This work was supported by the Schweizerischer Nationalfonds zur Förderung der Wissenschaftlichen Forschung, SciBase AB, and GenoSkin SAS.

## Conflicts of Interest

C.A. reports a patent application on “Methods and medical devices for analyzing epithelial barrier function.” P.S., C.A., and Y.M. report a patent for the technology titled “Methods and apparatus for measuring electrical impedance and assessing biological conditions of tissue samples.” P.S. declares receiving personal fees from SciBase AB. NG declares receiving personal fees from Genoskin SAS. C.A. has received research grants from the Swiss National Science Foundation, European Union (EU CURE, EU SynAir‐G), Novartis Research Institutes, (Basel, Switzerland), Stanford University (Redwood City, Calif), and SciBase (Stockholm, Sweden); is the Co‐Chair for EAACI Guidelines on Environmental Science in Allergic diseases and Asthma; is on the Advisory Boards of Sanofi/Regeneron (Bern, Switzerland, New York, USA), Stanford University Sean Parker Asthma Allergy Center (CA, USA), Novartis (Basel, Switzerland), Glaxo Smith Kline (Zurich, Switzerland), Bristol‐Myers Squibb (New York, USA), Seed Health (Boston, USA), and SciBase (Stockholm, Sweden); and is the Editor‐in‐Chief of Allergy. ML, HB, PD, CZ, CB, DY, YP, and JK have nothing to declare within the scope of this work.

## Supporting information


**Figure S1:** Extended Spearman correlation analysis of proteomic markers with 24‐h skin barrier impedance following SDS exposure. (A) Volcano plot of Spearman correlations between differentially expressed proteins (from proximity extension proteomics at 24 h post‐SDS treatment) and electrical impedance spectroscopy (EIS) values; points represent individual proteins, with red indicating significant positive correlations (Spearman's *ρ* > 0.5 and adjusted *p* < 0.05). (B) Scatter plots showing Spearman correlations for selected highlighted proteins (NPX values) vs. 24‐h EIS, with regression lines and individual ρ and *p*‐values indicated (ρ range: 0.587–0.762, all *p* < 0.05). Data are from skin lysates of NativeSkin treated with SDS or PBS for 5 min. Statistical significance: Spearman rank correlation analysis; *p*‐values are two‐tailed.
**Figure S2:** Protein membership of dose–response expression patterns. Proteins significantly altered at 48 h after 6 h of SDS exposure were grouped into four trajectory patterns as defined in Figure 2D. Each panel (A–D) shows the complete set of proteins within one pattern and their expression across SDS concentrations (0, 0.04, 0.2, 1, 5 mg/mL).
**Figure S3:** Correlation of protein expression in skin tissue lysates with EIS values. Scatter plots show correlations between EIS values measured 48 h after SDS exposure and protein expression levels (normalized protein expression; NPX, log2 scale) in skin tissue lysates at the same time point. For each plot, the protein name is indicated in the title, and the Spearman correlation coefficient (ρ) and *p*‐value are indicated.
**Figure S4:** Correlation of secreted protein levels in culture medium with EIS values. Scatter plots show correlations between EIS values measured at 48 h after SDS exposure and protein levels (normalized protein expression; NPX) in culture medium. For each plot, the protein name is indicated in the title, and the Spearman correlation coefficient (ρ) and *p*‐value are indicated.
**Figure S5:** Enrichment of downregulated pathways following SDS exposure. GO BP analysis identifies significantly downregulated pathways with the number of differentially expressed genes in the 0.05 mg/mL SDS‐treated group compared to the PBS control.
**Figure S6:** Cytotoxicity of NAC and NAM in keratinocytes. LDH release assays were performed in NHEK monolayers treated with increasing concentrations of N‐acetylcysteine (NAC) (A) or nicotinamide (NAM) (B) for 8 h. At the concentrations used in this study (2 mM NAC, 6 mM NAM), neither compound induced measurable cytotoxicity compared to PBS controls.
**Table S1:** Demographics of the ex vivo human skin donors. Donor information, including sex, age, Fitzpatrick skin type, body mass index (BMI), and anatomical site (abdomen). Donors were assigned to different experimental models (short‐term SDS exposure for 1 or 5 min, long‐term SDS exposure for 6 h, or antioxidant treatment with NAC or NAM).

## Data Availability

The data that support the findings of this study are openly available in Zenodo at https://doi.org/10.5281/zenodo.17432481, reference number doi:10.5281/zenodo.17432481.
