# Supplementary material for: Rapid Human Skin Barrier Disruption by Sodium Dodecyl Sulfate and Associated Molecular Mechanisms
Source: Allergy. 2026 May 17;81(7):2478–89. doi: 10.1111/all.70390 (PMC13342777; doi:10.1111/all.70390)
Supplement: Supplementary file 1 — Figure S1: Extended Spearman correlation analysis of proteomic markers with 24‐h skin barrier impedance following SDS exposure. (A) Volcano plot of Spearman correlations between differentially expressed proteins (from proximity extension proteomics at 24 h post‐SDS treatment) and electrical impedance spectroscopy (EIS) values; points represent individual proteins, with red indicating significant positive correlations (Spearman's ρ > 0.5 and adjusted p < 0.05). (B) Scatter plots showing Spearman correlations for selected highlighted proteins (NPX values) vs. 24‐h EIS, with regression lines and individual ρ and p‐values indicated (ρ range: 0.587–0.762, all p < 0.05). Data are from skin lysates of NativeSkin treated with SDS or PBS for 5 min. Statistical significance: Spearman rank correlation analysis; p‐values are two‐tailed. Figure S2: Protein membership of dose–response expression patterns. Proteins significantly altered at 48 h after 6 h of SDS exposure were grouped into four trajectory patterns as defined in Figure 2D. Each panel (A–D) shows the complete set of proteins within one pattern and their expression across SDS concentrations (0, 0.04, 0.2, 1, 5 mg/mL). Figure S3: Correlation of protein expression in skin tissue lysates with EIS values. Scatter plots show correlations between EIS values measured 48 h after SDS exposure and protein expression levels (normalized protein expression; NPX, log2 scale) in skin tissue lysates at the same time point. For each plot, the protein name is indicated in the title, and the Spearman correlation coefficient (ρ) and p‐value are indicated. Figure S4: Correlation of secreted protein levels in culture medium with EIS values. Scatter plots show correlations between EIS values measured at 48 h after SDS exposure and protein levels (normalized protein expression; NPX) in culture medium. For each plot, the protein name is indicated in the title, and the Spearman correlation coefficient (ρ) and p‐value are indicated. Figure S5: E [file ALL-81-2478-s001.docx]

Supplementary Materials for

**Sodium dodecyl sulfate triggers rapid human skin barrier**

Manru Li, Huseyn Babayev, Paolo D’Avino, Can Zeyneloğlu, Ceren Bicer, Duygu Yazici, Yagiz Pat, Per Svedenhag, Nicolas Gaudenzio, Cezmi A. Akdis, Yasutaka Mitamura

**Corresponding authors**

Yasutaka Mitamura and Cezmi A. Akdis, Swiss Institute of Allergy and Asthma Research (SIAF), University of Zurich, Herman-Burchard Strasse 9, CH-7265 Davos, Wolfgang, Switzerland.

Email: yasutaka.mitamura@siaf.uzh.ch and [cezmi.akdis@siaf.uzh.ch](mailto:cezmi.akdis@siaf.uzh.ch)

**
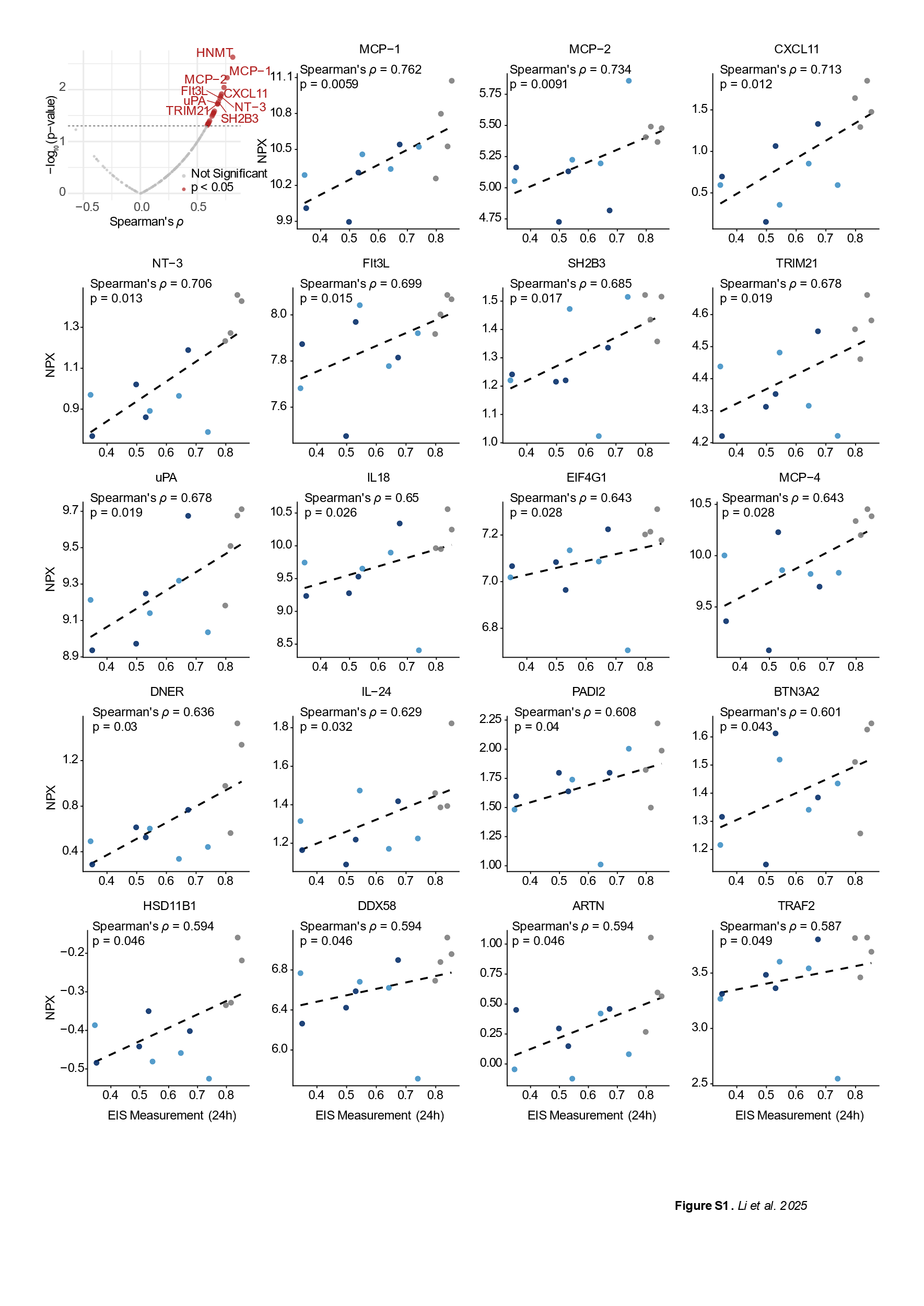
**

**Figure S1.** **Extended Spearman correlation analysis of proteomic markers with 24-hour skin barrier impedance following SDS exposure.** (**A**) Volcano plot of Spearman correlations between differentially expressed proteins (from proximity extension proteomics at 24 h post-SDS treatment) and electrical impedance spectroscopy (EIS) values; points represent individual proteins, with red indicating significant positive correlations (Spearman’s ρ > 0.5 and adjusted p < 0.05). (**B**) Scatter plots showing Spearman correlations for selected highlighted proteins (NPX values) versus 24-hour EIS, with regression lines and individual ρ and p-values indicated (ρ range: 0.587–0.762, all p < 0.05). Data are from skin lysates of NativeSkin® treated with SDS or PBS for 5 min. Statistical significance: Spearman rank correlation analysis; p-values are two-tailed.


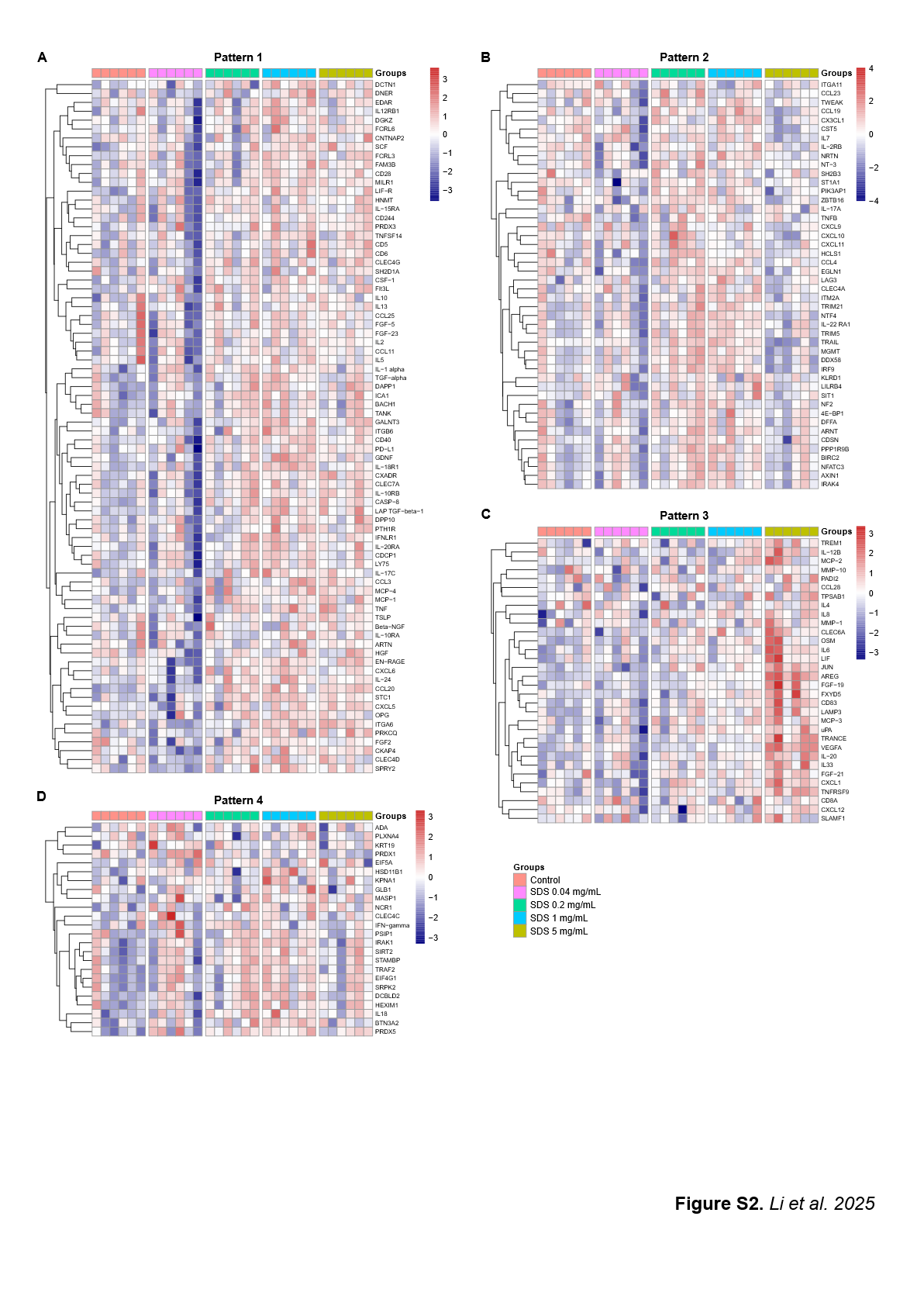


**Figure S2. Protein membership of dose–response expression patterns.** Proteins significantly altered at 48 h after 6 hours of SDS exposure were grouped into four trajectory patterns as defined in Figure 2D. Each panel (**A–D**) shows the complete set of proteins within one pattern and their expression across SDS concentrations (0, 0.04, 0.2, 1, 5 mg/mL).

**
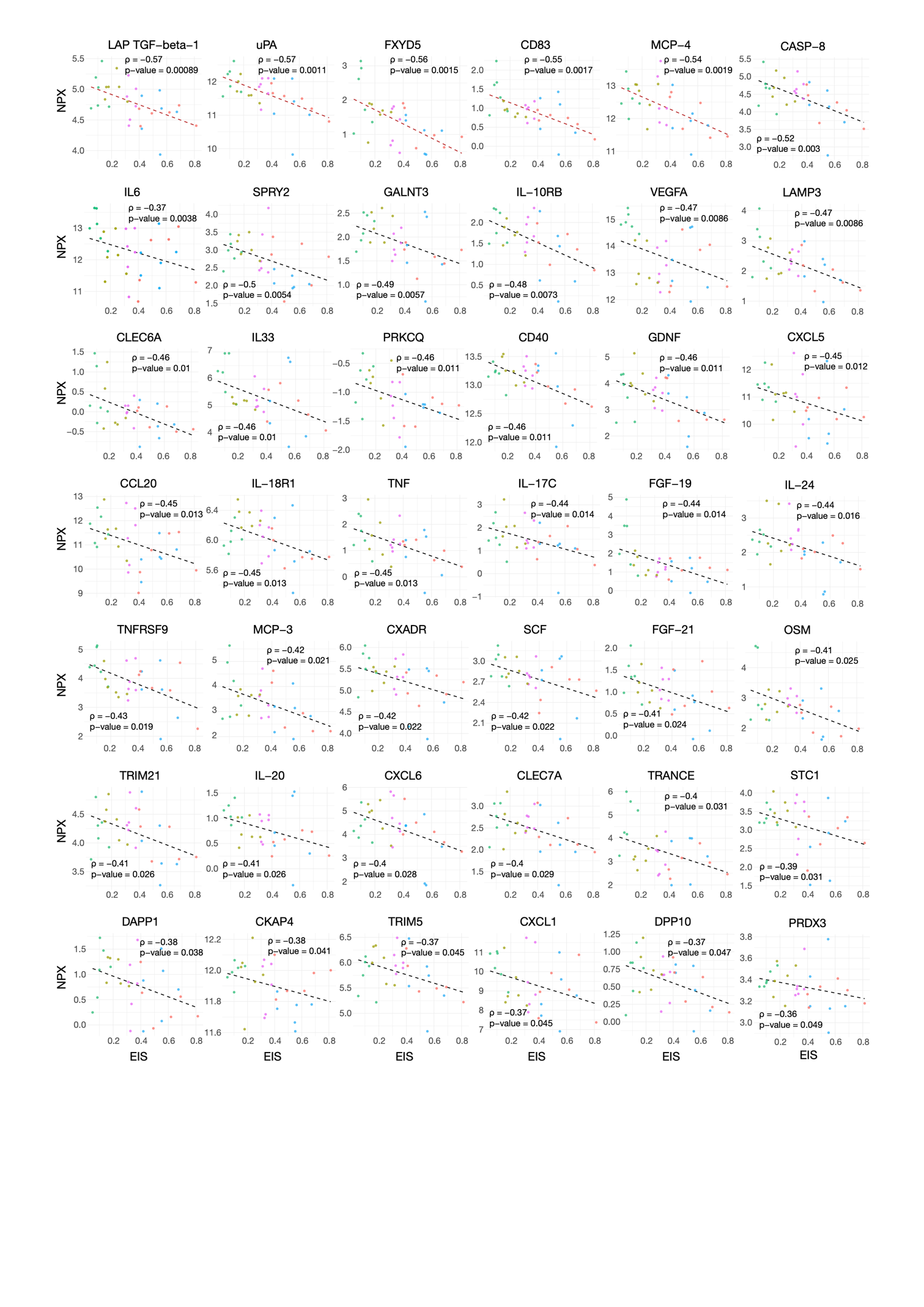
**

**Figure S3. Correlation of protein expression in skin tissue lysates with EIS values.** Scatter plots show correlations between EIS values measured 48 h after SDS exposure and protein expression levels (normalized protein expression; NPX, log2 scale) in skin tissue lysates at the same time point. For each plot, the protein name is indicated in the title, and the Spearman correlation coefficient (ρ) and p-value are indicated.

**Figure S4. Correlation of secreted protein levels in culture medium with EIS values.** Scatter plots show correlations between EIS values measured at 48 h after SDS exposure and protein levels (normalized protein expression; NPX) in culture medium. For each plot, the protein name is indicated in the title, and the Spearman correlation coefficient (ρ) and p-value are indicated.
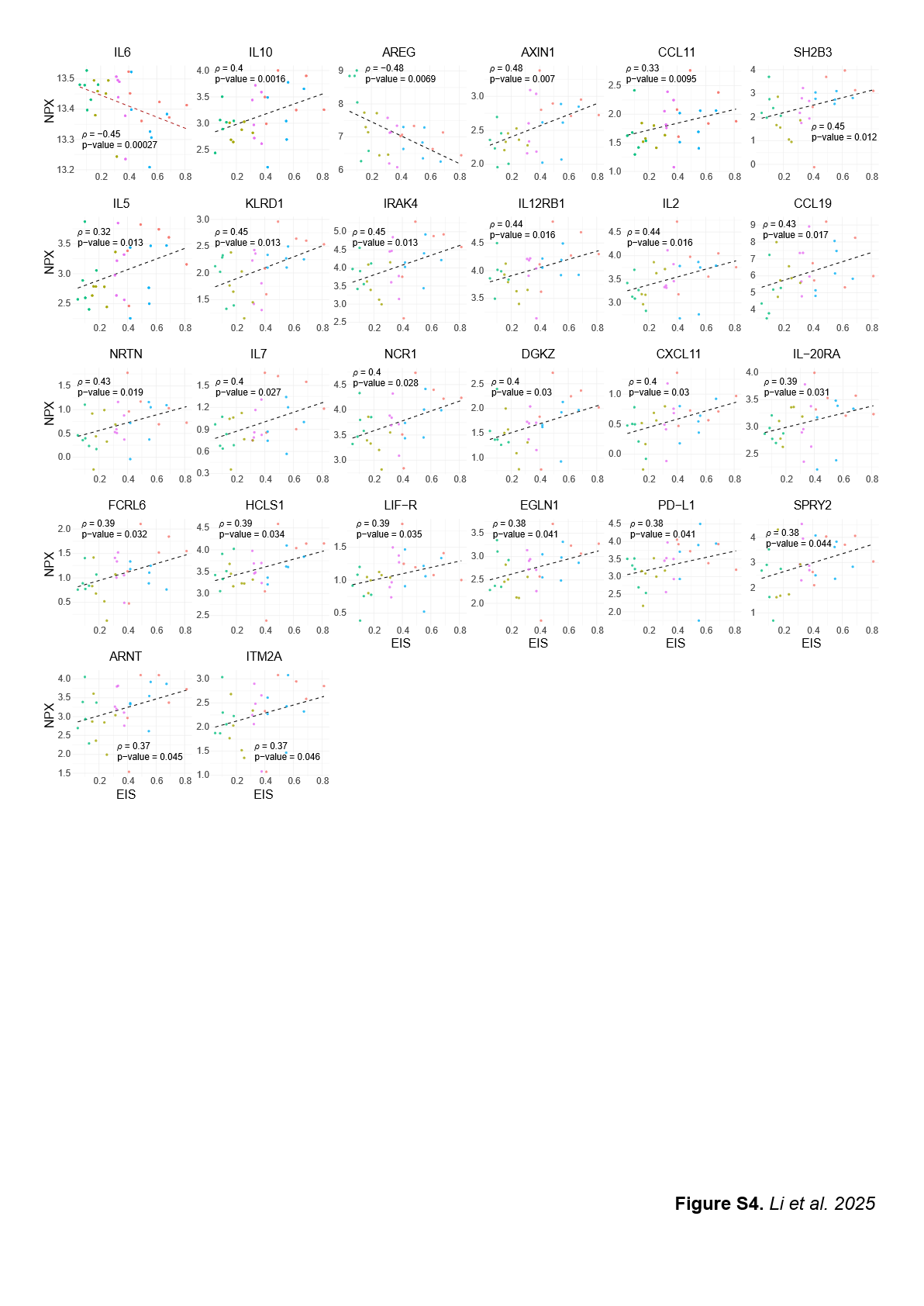


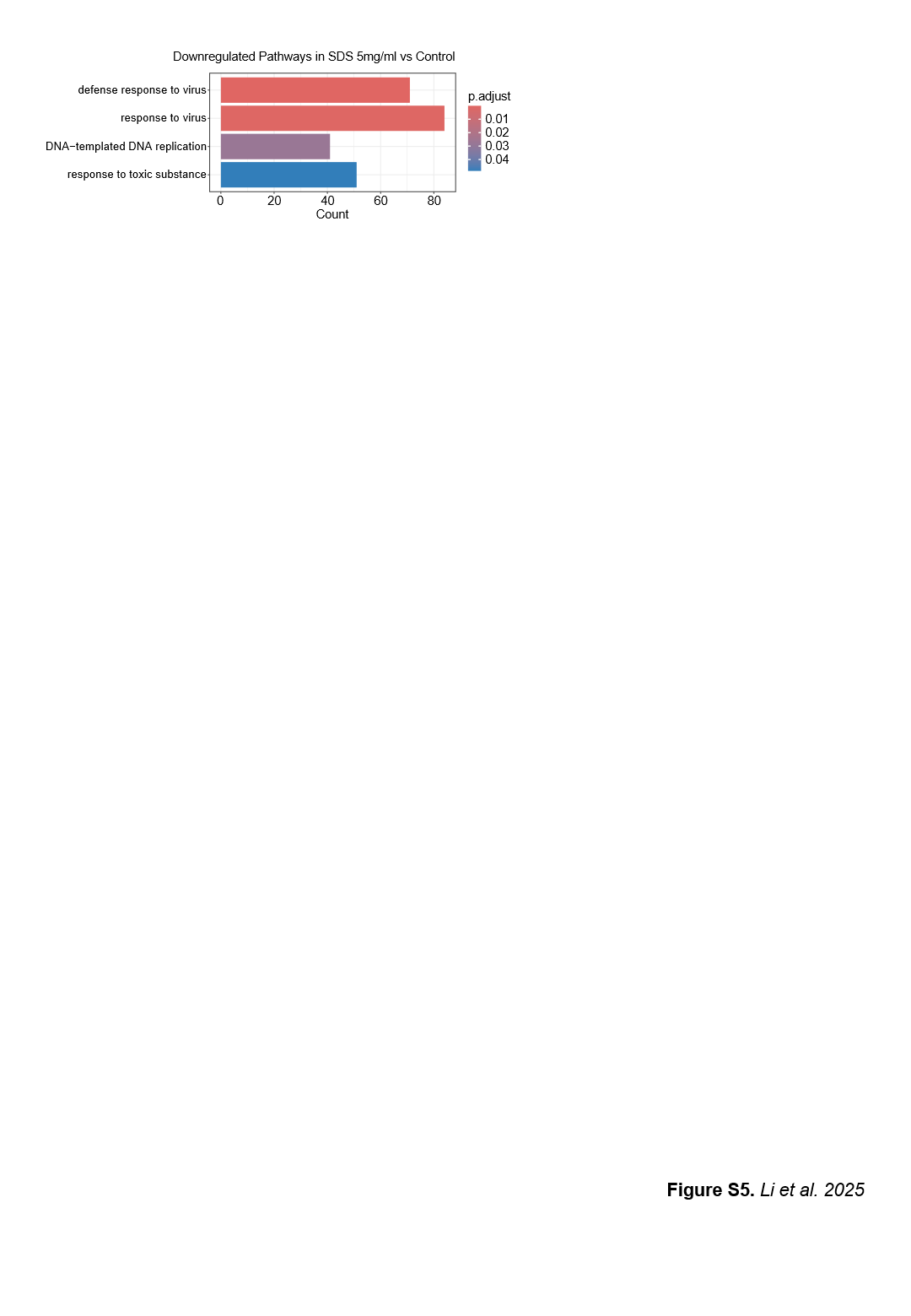


**Figure S5.** **Enrichment of downregulated pathways following SDS exposure.** GO BP analysis identifies significantly downregulated pathways with the number of differentially expressed genes in the 0.05 mg/mL SDS-treated group compared to the PBS control..


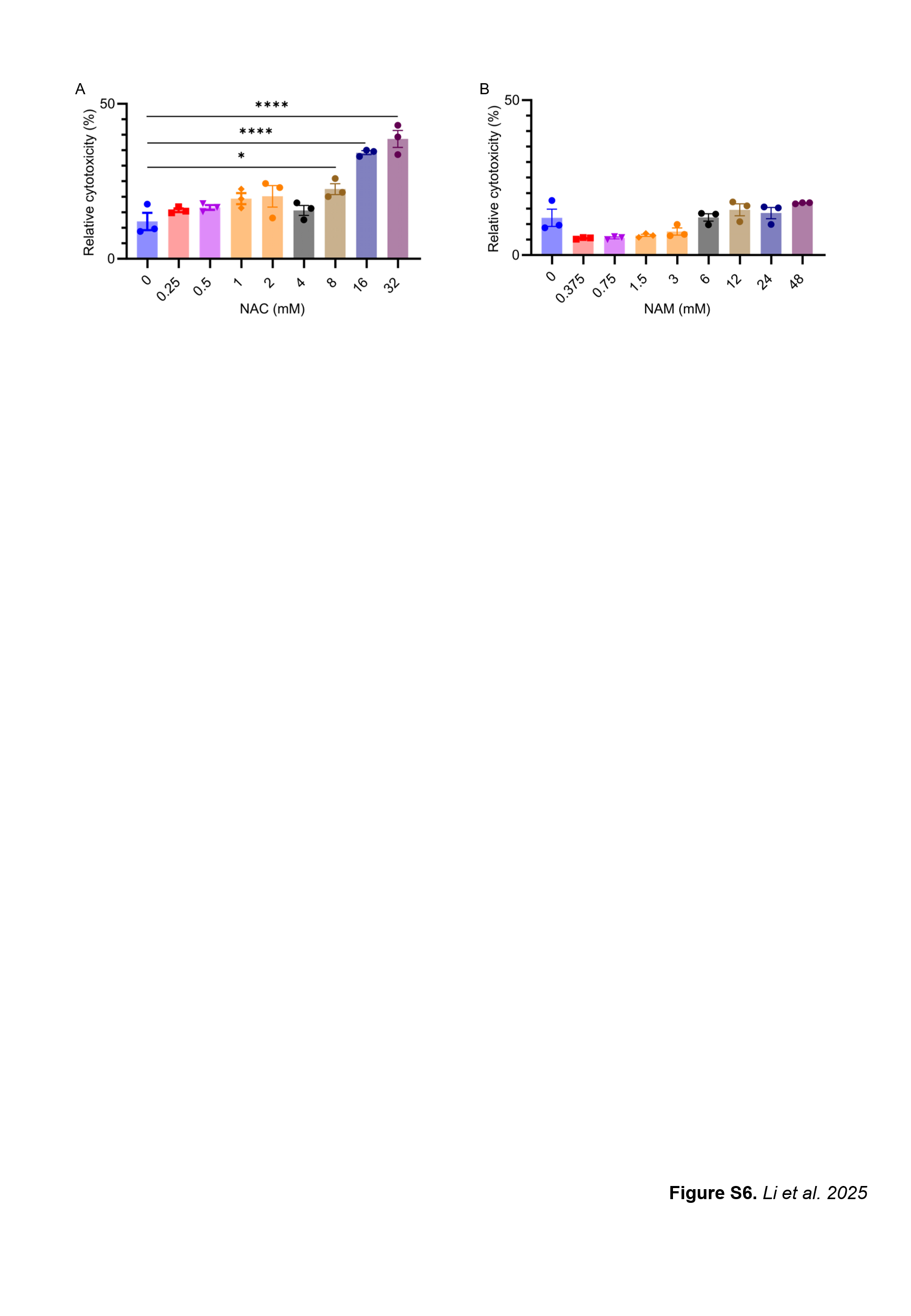


**Figure S6. Cytotoxicity of NAC and NAM in keratinocytes.** LDH release assays were performed in NHEK monolayers treated with increasing concentrations of N-acetylcysteine (NAC) (**A**) or nicotinamide (NAM) (**B**) for 8 h. At the concentrations used in this study (2 mM NAC, 6 mM NAM), neither compound induced measurable cytotoxicity compared to PBS controls.


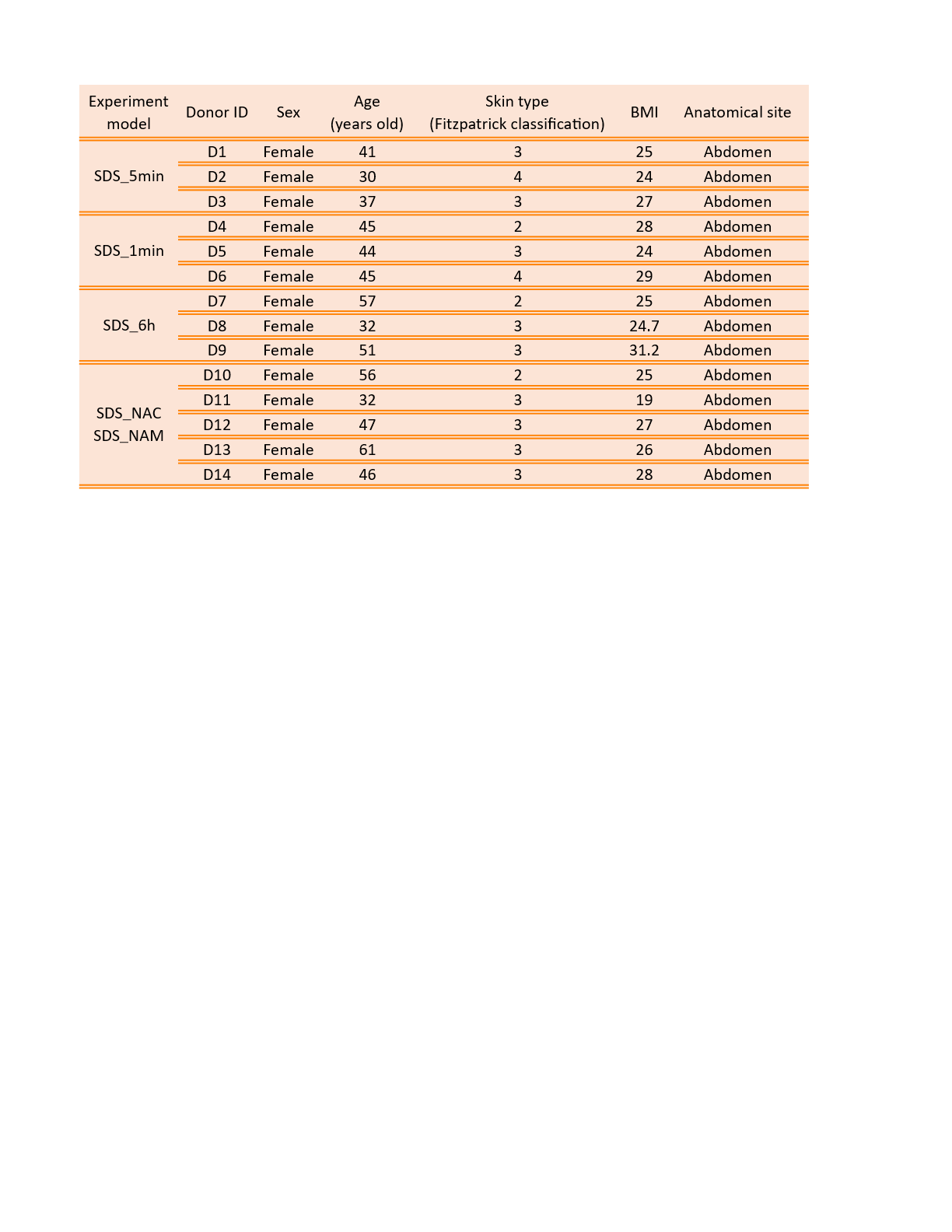


**Table S1. Demographics of the *ex vivo* human skin donors.** Donor information including sex, age, Fitzpatrick skin type, body mass index (BMI), and anatomical site (abdomen). Donors were assigned to different experimental models (short-term SDS exposure for 1 or 5 min, long-term SDS exposure for 6 h, or antioxidant treatment with NAC or NAM).
